# Association between metabolic syndrome components and impulse control disorders in Parkinson’s disease

**DOI:** 10.3389/fnins.2023.1191338

**Published:** 2023-05-18

**Authors:** Linxi Chen, Xinwei He, Taotao Tao, Linkao Chen, Yun Chen, Lingqun Mao, Peng Liu

**Affiliations:** ^1^Department of Neurology, Taizhou Central Hospital (Taizhou University Hospital), Taizhou, China; ^2^Department of Pathology, Taizhou Central Hospital (Taizhou University Hospital), Taizhou, China

**Keywords:** Parkinson’s disease, impulse control disorders (ICDs), compulsive eating, HbA1c, diabetes mellitus

## Abstract

**Background:**

Current evidence on management of impulse control disorders (ICDs) in Parkinson’s disease (PD) remains scarce, and exploring modifiable risk factors is crucial.

**Objective:**

We evaluated the profiles of ICDs in PD patients and aimed to determine the associations between ICDs, metabolic syndrome components and other clinical features.

**Methods:**

We enrolled patients diagnosed with PD in this study and conducted comprehensive clinical assessments.

**Results:**

We recruited 39 PD patients with ICDs and 66 PD patients without ICDs. Out of the 39 patients with ICDs, 19 (48.7%) had one impulse control disorder, while 20 (51.3%) had two or more. The most commonly reported symptom of ICDs was compulsive eating (48.7%). Significant differences were observed between the PD patients with and without ICDs in terms of their HbA1c levels, history of diabetes mellitus, dopamine agonist use, levodopa equivalent dose of dopamine agonists (LED DA), and Hamilton Depression Rating Scale (HAMD) scores. HbA1c levels were significantly higher in the PD patients with compulsive eating. Stepwise logistic regression analyses were performed with the dependent variables of ICDs (yes/no) and compulsive eating (yes/no). Among the 105 PD patients, those with ICDs exhibited higher levels of HbA1c, HAMD score and LED DA than those without ICDs (*p* < 0.01). Among 39 PD patients with ICDs, those with compulsive eating exhibited higher levels of HbA1c (OR = 2.148, 95% CI = 1.004–4.594, *p* < 0.05). Among 105 PD patients, those with compulsive eating exhibited higher levels of HbA1c, LED DA and HAMD score (*p* < 0.05).

**Conclusion:**

This study provides insights into the profiles of ICDs in PD patients and their associations with various clinical features. Compulsive eating was the most common ICDs symptom reported. Notably, HbA1c levels were found to be higher in patients with compulsive eating, indicating that poor blood glucose control may be a potential risk factor for ICDs in PD. However, it should be noted that the higher HbA1c levels could also be a consequence of compulsive eating rather than a causal factor for ICDs in PD. Further research is needed to confirm the modifiable risk factors for ICDs in PD.

## Introduction

Parkinson’s disease (PD) is typically recognized as a movement disorder, but it is often accompanied by a range of non-motor symptoms, such as neuropsychiatric symptoms, autonomic dysfunction, sleep disorders, and other conditions, which contribute to the overall burden of parkinsonian morbidity (Postuma et al., 2015; Schapira et al., 2017). Impulse control disorders (ICDs), such as pathological gambling, compulsive sexual behavior, compulsive shopping, and compulsive eating, are one of the non-motor symptoms that occur in PD (Voon et al., 2017; Weintraub, 2019). Other closely related phenomena include punding, hobbyism, hoarding, and dopamine dysregulation syndrome (Voon et al., 2017; Weintraub, 2019). ICDs are often neglected psychiatric complications in PD, yet they can have a significant impact on patients and their families (Antonini et al., 2017; Weintraub and Mamikonyan, 2019). However, current evidence on management of ICDs in PD remains scarce. The major management is to discontinue dopamine agonists (Weintraub and Mamikonyan, 2019). Few data support the use of neuropsychiatric drugs or behavioral interventions for ICDs in PD (Weintraub and Mamikonyan, 2019). The treatment of deep brain stimulation for ICDs in PD is also uncertain (Frank et al., 2007; Weintraub and Mamikonyan, 2019). Therefore, exploring modifiable risk factors and understanding how they link to ICDs in PD could allow for taking better treatment and care leading to reduced onset of ICDs and increased quality of life.

Interestingly, study has reported that ICDs were significantly associated with diabetes mellitus (DM) in a large sample of 52,095 community-dwelling adults from 19 countries (De Jonge et al., 2014). Moreover, data suggests a relationship between cholesterol and impulsivity when statistical analysis focuses on subjects with very low levels of cholesterol in healthy young men (Pozzi et al., 2003). On the other hand, metabolic syndrome components, including obesity, hypertension, DM and dyslipidemia have been reported to link to the risk of PD (Li et al., 2022; Schrag et al., 2023). It raises the question that whether metabolic syndrome components are related to ICDs in PD. To address this scientific question, we conducted a cross-sectional study to further explore the relationships between metabolic syndrome components and ICDs in PD.

## Methods

### Subjects

Patients diagnosed with PD according to the Movement Disorder Society clinical diagnostic criteria were enrolled in this cross-sectional study from January 2020 to February 2023 (Postuma et al., 2015). Exclusion criteria included atypical clinical features, dementia, severe psychosis, malignancies, a use of a dopamine receptor blocking agent, or previous PD neurosurgery. The study was approved by the Ethics Committee of Taizhou Central Hospital. Written informed consent was obtained according to the Declaration of Helsinki.

### Clinical assessments

The patients’ demographic data and clinical features, such as age, sex, disease duration, level of education, body mass index, medical history, and details of current therapies were collected. Testing of Hemoglobin A1c (HbA1c) level was used to reflect the recent blood glucose control status. The modified Hoehn and Yahr staging scale (H&Y) was used to evaluate disease severity (Goetz et al., 2004). Mini-Mental State Examination was used to estimate the general cognitive function (Folstein et al., 1975). The 24-item Hamilton Depression Rating Scale (HAMD) was used to estimate the severity of depression (Williams, 2001). ICDs was defined by Questionnaire for Impulsive-Compulsive Disorders in Parkinson’s Disease (QUIP-Current-Short version) (Weintraub et al., 2009).

### Statistical analysis

The Kolmogorov–Smirnov test was used for normality testing. Continuous variables were presented as the mean ± SD, while categorical data were presented as frequencies (percentages) or medians and interquartile ranges. Comparisons between two groups were conducted using the independent *t*-test, Mann–Whitney *U*-test, or Fisher’s exact test, as appropriate. Considering the sample sizes for gambling, sex, and buying behaviors in our study were too small to meet the minimum requirements for statistical power. Therefore, we only showed the between-group comparison of PD patients with and without compulsive eating. Forward stepwise logistic regression analyses were performed with group status as dependent variable, the variable of interest as independent variable. Multiple linear regression analyses were performed for diagnosing of multicollinearity for each candidate variate. In our analysis of Model A and C, we included age, sex, BMI, H&Y, level of education, disease duration, and MMSE as potential factors related to ICDs, even though they did not reach statistical significance. For model B, considering the small statistical sample size (n = 39), we limit the number of variables in the multivariable analysis. Therefore, BMI, level of education, disease duration, and MMSE were not analyzed in Model B. Two-tailed *p-*values were calculated for all analyses. The alpha level of significance was set at 0.05. All analyses were performed using SPSS version 25 (IBM Corp., Armonk, NY, United States).

## Results

### Demographic data and clinical features

The demographic and clinical characteristics of all participants were presented in Table 1. Thirty-nine PD patients with ICDs (18 females and 21 males) and 66 PD patients without ICDs (30 females and 36 males) were recruited to the study. The mean age was 68.4 ± 3.1 years, with a mean disease duration of 4.9 ± 1.5 years in PD with ICDs group. The mean age was 68.3 ± 2.9 years, with a mean disease duration of 5.3 ± 1.2 years in PD without ICDs group. There were significant differences between the PD with ICDs group and PD without ICDs group in HbA1c, history of DM, dopamine agonist use, levodopa equivalent dose of dopamine agonist (LED DA), and HAMD score. There were no significant differences between the PD with ICDs group and PD without ICDs group in age, sex, disease duration, level of education, body mass index, medical history (smoking, alcohol, dyslipidemia, and hypertension).

**Table 1:** Demographic data and clinical features in Parkinson’s disease patients.

	ICDs group (*n* = 39)	Non-ICDs group (*n* = 66)	*p* value
Age (years)	68.4 ± 3.1	68.3 ± 2.9	n.s.
Female, *n* (%)	18 (46.2)	30 (45.5)	n.s.
Disease duration (years)	4.9 ± 1.5	5.3 ± 1.2	n.s.
Education, *n* (%)			n.s.
No education to secondary School	28 (71.8)	53 (80.3)	
High school	9 (23.1)	7 (10.6)	
University	2 (5.1)	6 (9.1)	
BMI (kg/m^2^)	24.2 ± 2.6	24.4 ± 3.1	n.s.
Smoking, *n* (%)	15 (38.5)	30 (45.5)	n.s.
Alcohol, *n* (%)	14 (35.9)	34 (51.5)	n.s.
Dyslipidemia, *n* (%)	7 (17.9)	12 (18.2)	n.s.
Hypertension, *n* (%)	7 (17.9)	14 (21.2)	n.s.
Diabetes, *n* (%)	16 (41)	10 (15.2)	0.005^**^
HbA1c (%)	6.0 ± 1.2	5.0 ± 0.8	<0.001^**^
Hoehn and Yahr stage	2 (2–2.5)	2 (2–2.5)	n.s.
Levodopa use, *n* (%)	31 (79.5)	55 (83.3)	n.s.
LED levodopa (mg)	234.6 ± 134.8	278.0 ± 157.7	n.s.
DA use, *n* (%)	31 (79.5)	39 (59.1)	0.035^*^
LED DA (mg)	168.6 ± 93.5	100.76 ± 94.4	0.001^**^
COMT use, *n* (%)	13 (33.3)	23 (34.8)	n.s.
MAO-B use, *n* (%)	21 (53.8)	40 (60.6)	n.s.
Amantadine use, *n* (%)	4 (10.3)	8 (12.1)	n.s.
Benzhexol use, *n* (%)	2 (5.1)	4 (6.1)	n.s.
MMSE	24.3 ± 3.3	23.4 ± 3.0	n.s.
HAMD	13.2 ± 7.9	7.5 ± 4.7	<0.001^**^

ICDs, impulse control disorders; BMI, body mass index; LED, levodopa equivalent dose; DA, dopamine agonist; COMT, catechol-o-methyltransferase inhibitors; MAO-B, monoamine oxidase-b inhibitors; MMSE, Mini-Mental State Examination; HAMD, Hamilton Depression Scale; n.s., not significant; *p < 0.05; **p < 0.01.

### ICDs profile in PD

**Figure 1:**
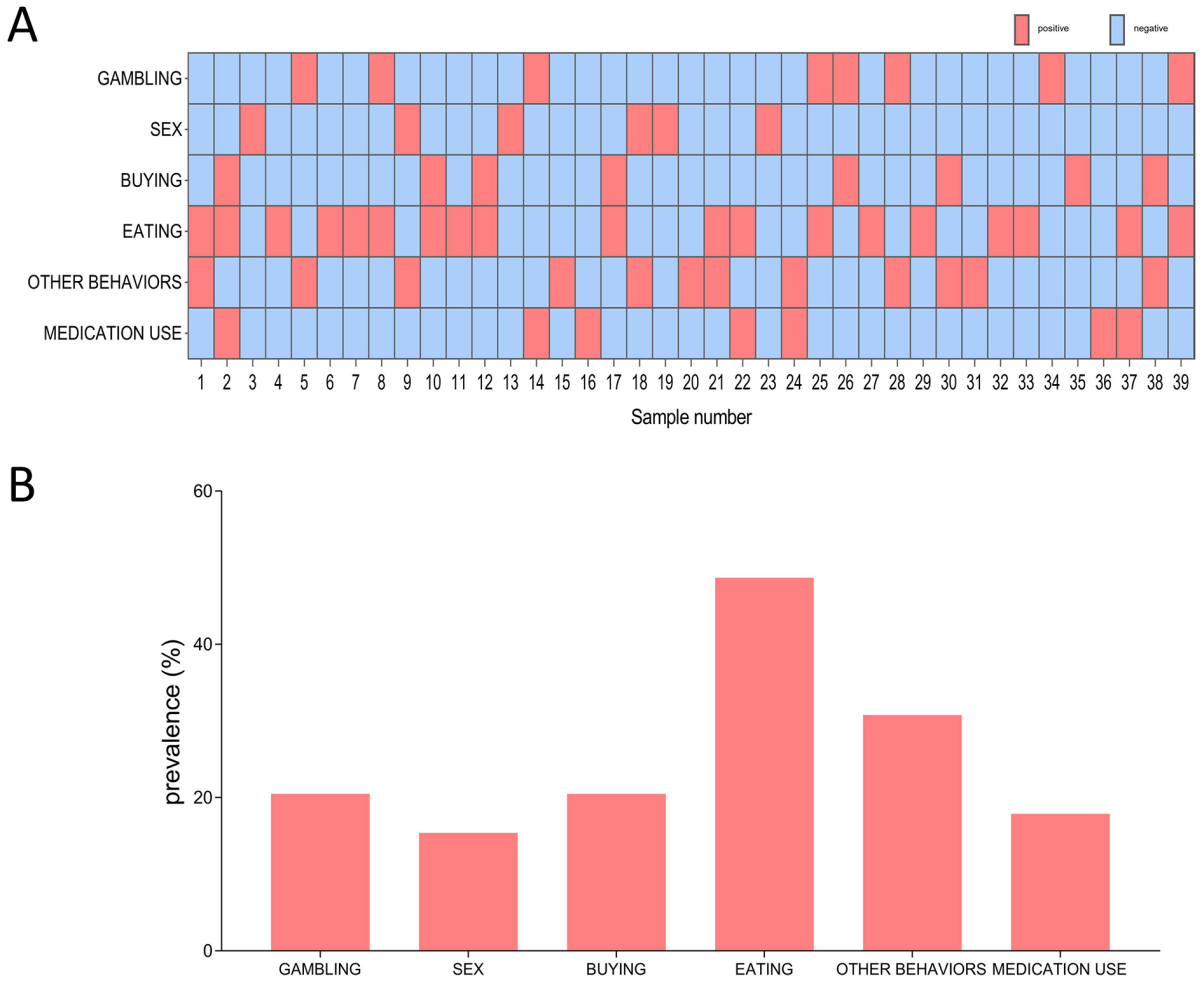
The distribution and frequency of ICDs among 39 QUIP positive PD patients. **(A)** Distribution of different types of ICDs symptom. Red box indicates positive for a certain type of ICDs, blue box indicates negative for certain type of ICDs. **(B)** Frequency of different types ICDs symptom.

Out of the 39 QUIP positive PD patients, 19 (48.7%) had one type of ICDs symptoms, and 20 (51.3%) had two or more types of ICDs symptoms (Figure 1A). Regarding QUIP-Current-Short part A to F (Figure 1B), compulsive eating (*n* = 19, 48.7%) was the most frequently reported ICDs symptom, followed by other behaviors (30.8%), gambling (20.5%), shopping (20.5%), medication use (17.9%) and sex (15.4%).

**Figure 2:**
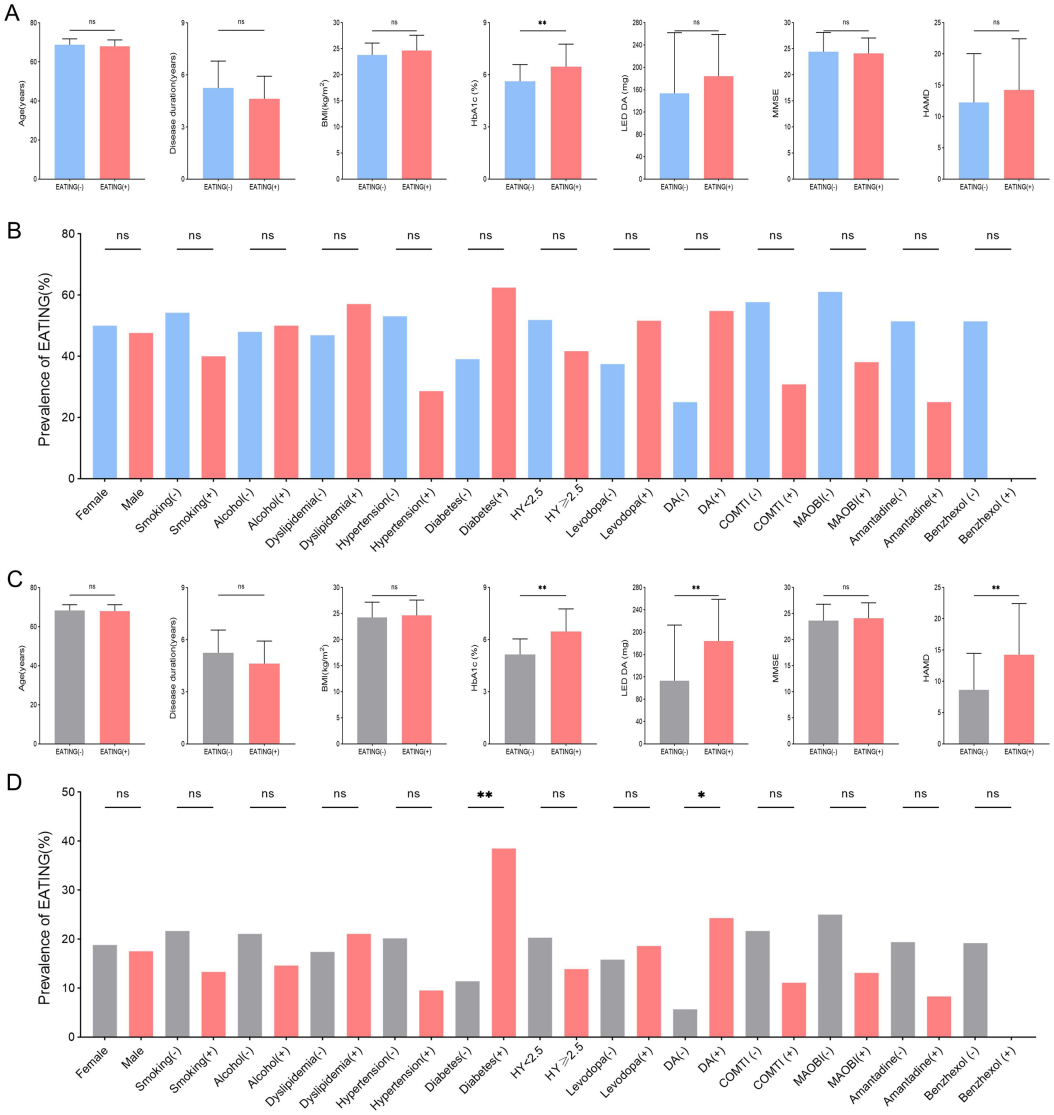
Between-group comparison of PD patients with compulsive eating (EATING +) and without compulsive eating (EATING -). **(A,B)** Among 39 QUIP positive PD patients, those with compulsive eating (EATING +) had substantially higher HbA1c levels (*p* = 0.026) than those without compulsive eating (EATING-). **(C,D)** Among 105 PD patients, there were significant differences between PD with compulsive eating (EATING +) and PD without compulsive eating (EATING +) in HbA1c levels (*p* < 0.001), LED DA (*p* = 0.001), history of DM (*p* = 0.006), dopamine agonist use (*p* = 0.029), and HAMD score (*p* = 0.001).

Among 39 QUIP positive PD patients, those with compulsive eating (EATING+) had substantially higher HbA1c levels (*p* = 0.026) than those without compulsive eating (EATING −) (Figures 2A,B). Among 105 PD patients, there were significant differences between PD with compulsive eating (EATING +) and PD without compulsive eating (EATING +) in HbA1c levels (*p* < 0.001), LED DA (*p* = 0.001), history of DM (*p* = 0.006), dopamine agonist use (*p* = 0.029), and HAMD score (*p* = 0.001) (Figures 2C,D).

### Multivariable analysis to explore correlative factors of ICDs in PD

Data from the stepwise logistic regression were shown in Table 2. Among the 105 PD patients, those with ICDs exhibited higher levels of HbA1c, HAMD score and LED DA than those without ICDs (*p* < 0.01, model A of Table 2). Among 39 QUIP positive PD patients, those with compulsive eating exhibited higher levels of HbA1c (OR = 2.148, 95% CI = 1.004–4.594, *p* < 0.05, model B of Table 2). Among 105 PD patients, those with compulsive eating exhibited higher levels of HbA1c, LED DA and HAMD score (*p* < 0.05, model C of Table 2).

**Table 2:** Analysis for the correlative factors of ICDs in PD patients.

	Dependent variable	Independent signifcant covariates	OR	95% CI	*p* value
Model A	ICDs (yes/no)	HbA1c (%)	2.397	1.276–4.502	0.007
		HAMD	1.159	1.057–1.272	0.002
		LED DA	1.007	1.002–1.013	0.007
		Age			0.542
		Sex			0.781
		BMI			0.167
		HY			0.705
		Education			0.762
		Disease duration			0.910
		MMSE			0.104
Model B	EATING (yes/no)	HbA1c (%)	2.148	1.004–4.594	0.049
		Age			0.164
		Sex			0.990
		HY			0.552
		LED DA			0.463
		HAMD			0.651
Model C	EATING (yes/no)	HbA1c (%)	2.119	1.256–3.575	0.005
		LED DA	1.008	1.001–1.015	0.034
		HAMD	1.118	1.022–1.224	0.015
		Age			0.293
		Sex			0.977
		BMI			0.877
		HY			0.312
		Education			0.658
		Disease duration			0.210
		MMSE			0.409

In Model A, we conducted a multivariable analysis to explore the correlative factors of ICDs in PD patients (*n* = 105). In Model B, we conducted a multivariable analysis to explore the correlative factors of compulsive eating in PD patients with ICDs (*n* = 39). In Model C, we conducted a multivariable analysis to explore the correlative factors of compulsive eating in PD patients (*n* = 105). PD, Parkinson’s disease; ICDs, impulse control disorders; EATING, compulsive eating; OR, odds ratio; CI: confidence interval; BMI, body mass index; HY, Hoehn and Yahr stage; HAMD, Hamilton Depression Scale; MMSE, Mini-Mental State Examination; LED DA, levodopa equivalent dose of dopamine agonist.

In model A, B and C, all independent variables’ tolerance was more than 0.2 and variance inflation factor was less than 3 (Supplementary Table S1), suggesting there was no multicollinearity in the model. The Hosmer and Lemeshow test revealed that the model has a high goodness of fit (*p* > 0.2).

## Discussion

The patients included in this study are all late-onset PD patients, with an elevated average age, who suffer from both motor and non-motor symptoms, including ICDs, depression, and cognitive impairment, which are related to PD, along with various components of metabolic syndrome. This may result in a greater symptom burden of these metabolic factors in these patients, which in turn further affects PD symptoms and creates a vicious cycle.

Consistent with prior research (Marin-Lahoz et al., 2019), our study indicates that depression and dopamine agonist use are both independently associated with ICDs in PD. It has been historically believed that serotoninergic pathways are an essential factor in the development of depression (Cowen, 2008). Genetic variation in these pathways also contribute to the susceptibility to ICDs in PD (Lee et al., 2012; Kraemmer et al., 2016). It indicates potential association between these two conditions. Additional research is required to determine if treating depression effectively can improve ICDs in PD. Generally, when treating patients with PD who also suffer from depression, pramipexole is often used by clinicians due to its demonstrated efficacy in addressing both motor symptoms and depression in PD patients (Rektorova et al., 2003; Barone et al., 2010). However, the combination of these two risk factors may increase the likelihood of developing ICDs (Marin-Lahoz et al., 2019). Therefore, it is crucial to carefully consider treatment options and inform patients of the potential risks.

Our data suggest that there was a correlation between HbA1c levels and ICDs in PD patients. Further analysis of subgroups revealed a significant association between compulsive eating and HbA1c levels. These findings suggest that inadequate management of blood glucose levels may increase the risk of ICDs in patients PD and contribute to a higher prevalence of compulsive eating among PD patients with ICDs. However, the nature of the relationship between inadequate management of blood glucose levels, ICDs and PD remains unclear. Studies has shown that ICDs are significantly associated with DM, and compulsive eating is independently linked with higher HbA1c in DM patients (De Jonge et al., 2014; Huisman et al., 2023). Growing evidence suggests that the biological mechanisms and pathways responsible for DM at the cellular level could potentially trigger or interact with pathways involved in the development of PD. Studies have revealed that patients with T2DM who do not have PD display indications of subclinical striatal dopaminergic dysfunction (Pagano et al., 2018). Likewise, experiments on healthy mice that were fed a high-fat diet to induce peripheral insulin resistance have shown nigrostriatal dopaminergic dysfunction and parkinsonism, indicating a connection between the pathophysiology of PD and T2DM (Wu et al., 2019). It is highly probable that these two medical conditions are linked through dysregulated pathophysiological pathways, which in turn form the basis for the development of ICDs, such as compulsive eating (Athauda et al., 2022). One hypothesis is that blood glucose levels may potentially contribute to eating disorders and subsequent ICDs. This needs to be clarified by further longitudinal studies. On the other hand, PD can affect patients’ mobility, leading to a decrease in physical activity levels, which can make it difficult for the body to effectively utilize glucose. Moreover, PD can affect patient’s dietary changes to adjust their diet according to their condition, such as reducing protein intake and frequently changing eating time. These conditions may lead to poor blood glucose control and further provide the foundation for ICDs in PD patients.

We acknowledge that our study’s cross-sectional design limits our ability to establish causality between poor blood glucose control, ICDs, compulsive eating and PD. Given the complex interplay among these variables, the associations we observed are likely bidirectional and may vary over time. Thus, future longitudinal research is necessary to establish a causal relationship between these variables and better understand their dynamic nature. Only then can we draw more definitive conclusions about the factors that contribute to the development and maintenance of ICDs, and their impact on physical and mental health outcomes.

In summary, this study provides insights into the profile of ICDs in PD patients and their associations with various clinical features. The most common symptom reported was compulsive eating. Notably, HbA1c levels are found to be higher in patients with compulsive eating, indicating that poor blood glucose control may be a potential risk factor for ICDs in PD. However, it should be noted that the higher HbA1c levels could also be a consequence of compulsive eating rather than a causal factor for ICDs in PD. The findings highlight the need for further research to confirm the modifiable risk factors for ICDs in PD and to develop effective management strategies for this condition.

## Data availability statement

The original contributions presented in the study are included in the article/Supplementary material, further inquiries can be directed to the corresponding authors.

## Ethics statement

The studies involving human participants were reviewed and approved by Taizhou Central Hospital. The patients/participants provided their written informed consent to participate in this study.

## Author contributions

PL, LM, and LXC: conception and organization of research project. TT, LKC, YC, and XH: execution of the research project. PL and XH: statistical analysis design and execution. LXC: manuscript writing of the first draft. LM, PL, and XH: manuscript review and critique. All authors contributed to the article and approved the submitted version.

## Conflict of interest

The authors declare that the research was conducted in the absence of any commercial or financial relationships that could be construed as a potential conflict of interest.

## Publisher’s note

All claims expressed in this article are solely those of the authors and do not necessarily represent those of their affiliated organizations, or those of the publisher, the editors and the reviewers. Any product that may be evaluated in this article, or claim that may be made by its manufacturer, is not guaranteed or endorsed by the publisher.

## References

[ref1] Antonini A. Barone P. Bonuccelli U. Annoni K. Asgharnejad M. Stanzione P. (2017). ICARUS study: prevalence and clinical features of impulse control disorders in Parkinson's disease. J. Neurol. Neurosurg. Psychiatry 88, 317–324. doi: 10.1136/jnnp-2016-315277, PMID: 28315845

[ref2] Athauda D. Evans J. Wernick A. Virdi G. Choi M. L. Lawton M.. (2022). The impact of type 2 diabetes in Parkinson's disease. Mov. Disord. 37, 1612–1623. doi: 10.1002/mds.29122, PMID: 35699244 PMC9543753

[ref3] Barone P. Poewe W. Albrecht S. Debieuvre C. Massey D. Rascol O.. (2010). Pramipexole for the treatment of depressive symptoms in patients with Parkinson's disease: a randomised, double-blind, placebo-controlled trial. Lancet Neurol. 9, 573–580. doi: 10.1016/S1474-4422(10)70106-X, PMID: 20452823

[ref4] Cowen P. J. (2008). Serotonin and depression: pathophysiological mechanism or marketing myth? Trends Pharmacol. Sci. 29, 433–436. doi: 10.1016/j.tips.2008.05.004 18585794

[ref5] De Jonge P. Alonso J. Stein D. J. Kiejna A. Aguilar-Gaxiola S. Viana M. C.. (2014). Associations between DSM-IV mental disorders and diabetes mellitus: a role for impulse control disorders and depression. Diabetologia 57, 699–709. doi: 10.1007/s00125-013-3157-9, PMID: 24488082 PMC4124905

[ref6] Folstein M. F. Folstein S. E. Mchugh P. R. (1975). "Mini-mental state". A practical method for grading the cognitive state of patients for the clinician. J. Psychiatr. Res. 12, 189–198. doi: 10.1016/0022-3956(75)90026-6 1202204

[ref7] Frank M. J. Samanta J. Moustafa A. A. Sherman S. J. (2007). Hold your horses: impulsivity, deep brain stimulation, and medication in parkinsonism. Science 318, 1309–1312. doi: 10.1126/science.1146157, PMID: 17962524

[ref8] Goetz C. G. Poewe W. Rascol O. Sampaio C. Stebbins G. T. Counsell C.. (2004). Movement Disorder Society task force report on the Hoehn and Yahr staging scale: status and recommendations. Mov. Disord. 19, 1020–1028. doi: 10.1002/mds.20213 15372591

[ref9] Huisman S. D. Hendrieckx C. Bot M. Pouwer F. Nefs G. (2023). Prevalence, associations and health outcomes of binge eating in adults with type 1 or type 2 diabetes: results from diabetes MILES - the Netherlands. Diabet. Med. 40:e14953. doi: 10.1111/dme.14953 36084309 PMC10087813

[ref10] Kraemmer J. Smith K. Weintraub D. Guillemot V. Nalls M. A. Cormier-Dequaire F.. (2016). Clinical-genetic model predicts incident impulse control disorders in Parkinson's disease. J. Neurol. Neurosurg. Psychiatry 87, 1106–1111. doi: 10.1136/jnnp-2015-312848, PMID: 27076492 PMC5098340

[ref11] Lee J. Y. Jeon B. S. Kim H. J. Park S. S. (2012). Genetic variant of HTR2A associates with risk of impulse control and repetitive behaviors in Parkinson's disease. Parkinsonism Relat. Disord. 18, 76–78. doi: 10.1016/j.parkreldis.2011.08.009, PMID: 21900033

[ref12] Li L. Y. Liu S. F. Zhuang J. L. Li M. M. Huang Z. P. Chen Y. H.. (2022). Recent research progress on metabolic syndrome and risk of Parkinson's disease. Rev. Neurosci. doi: 10.1515/revneuro-2022-0093, PMID: 36450297

[ref13] Marin-Lahoz J. Sampedro F. Martinez-Horta S. Pagonabarraga J. Kulisevsky J. (2019). Depression as a risk factor for impulse control disorders in Parkinson disease. Ann. Neurol. 86, 762–769. doi: 10.1002/ana.25581, PMID: 31415102

[ref14] Pagano G. Polychronis S. Wilson H. Giordano B. Ferrara N. Niccolini F.. (2018). Diabetes mellitus and Parkinson disease. Neurology 90, e1654–e1662. doi: 10.1212/WNL.0000000000005475 29626177

[ref15] Postuma R. B. Berg D. Stern M. Poewe W. Olanow C. W. Oertel W.. (2015). MDS clinical diagnostic criteria for Parkinson's disease. Mov. Disord. 30, 1591–1601. doi: 10.1002/mds.26424 26474316

[ref16] Pozzi F. Troisi A. Cerilli M. Autore A. M. Lo Castro C. Ribatti D.. (2003). Serum cholesterol and impulsivity in a large sample of healthy young men. Psychiatry Res. 120, 239–245. doi: 10.1016/S0165-1781(03)00192-6, PMID: 14561435

[ref17] Rektorova I. Rektor I. Bares M. Dostal V. Ehler E. Fanfrdlova Z.. (2003). Pramipexole and pergolide in the treatment of depression in Parkinson's disease: a national multicentre prospective randomized study. Eur. J. Neurol. 10, 399–406. doi: 10.1046/j.1468-1331.2003.00612.x, PMID: 12823492

[ref18] Schapira A. H. V. Chaudhuri K. R. Jenner P. (2017). Non-motor features of Parkinson disease. Nat. Rev. Neurosci. 18, 435–450. doi: 10.1038/nrn.2017.62 28592904

[ref19] Schrag A. Bohlken J. Dammertz L. Teipel S. Hermann W. Akmatov M. K.. (2023). Widening the Spectrum of risk factors, comorbidities, and prodromal features of Parkinson disease. JAMA Neurol. 80, 161–171. doi: 10.1001/jamaneurol.2022.3902, PMID: 36342675 PMC9641600

[ref20] Voon V. Napier T. C. Frank M. J. Sgambato-Faure V. Grace A. A. Rodriguez-Oroz M.. (2017). Impulse control disorders and levodopa-induced dyskinesias in Parkinson's disease: an update. Lancet Neurol. 16, 238–250. doi: 10.1016/S1474-4422(17)30004-2, PMID: 28229895

[ref21] Weintraub D. (2019). Impulse control disorders in Parkinson's disease: a 20-year odyssey. Mov. Disord. 34, 447–452. doi: 10.1002/mds.27668, PMID: 30913337

[ref22] Weintraub D. Hoops S. Shea J. A. Lyons K. E. Pahwa R. Driver-Dunckley E. D.. (2009). Validation of the questionnaire for impulsive-compulsive disorders in Parkinson's disease. Mov. Disord. 24, 1461–1467. doi: 10.1002/mds.22571, PMID: 19452562 PMC2848971

[ref23] Weintraub D. Mamikonyan E. (2019). Impulse control disorders in Parkinson's disease. Am. J. Psychiatry 176, 5–11. doi: 10.1176/appi.ajp.2018.18040465 30848949

[ref24] Williams J. B. (2001). Standardizing the Hamilton depression rating scale: past, present, and future. Eur. Arch. Psychiatry Clin. Neurosci. 251:II6-12. doi: 10.1007/BF03035120, PMID: 11824839

[ref25] Wu H. Xie B. Ke M. Deng Y. (2019). High-fat diet causes increased endogenous neurotoxins and phenotype of Parkinson's disease in mice. Acta Biochim. Biophys. Sin. 51, 969–971. doi: 10.1093/abbs/gmz073, PMID: 31392317

