# Supplementary material for: Association between metabolic syndrome components and impulse control disorders in Parkinson’s disease
Source: Front Neurosci. 2023 May 18;17:1191338. doi: 10.3389/fnins.2023.1191338 (PMC10232783; doi:10.3389/fnins.2023.1191338)
Supplement: Supplementary file 1 [file Table_1.DOCX]

**Supplementary Table 1** Multiple linear regression for diagnosing of multicollinearity for each candidate variate

|  | Model A | | Model B | | Model C | |
| --- | --- | --- | --- | --- | --- | --- |
|  | Tolerance | VIF | Tolerance | VIF | Tolerance | VIF |
| Age | 0.839 | 1.191 | 0.776 | 1.289 | 0.839 | 1.191 |
| Sex | 0.868 | 1.152 | 0.902 | 1.108 | 0.868 | 1.152 |
| HY | 0.831 | 1.203 | 0.806 | 1.241 | 0.831 | 1.203 |
| HbA1c | 0.775 | 1.291 | 0.868 | 1.152 | 0.775 | 1.291 |
| LED DA | 0.851 | 1.175 | 0.902 | 1.108 | 0.851 | 1.175 |
| HAMD | 0.854 | 1.171 | 0.640 | 1.562 | 0.854 | 1.171 |
| Education | 0.789 | 1.267 | - | - | 0.789 | 1.267 |
| Disease duration | 0.800 | 1.250 | - | - | 0.800 | 1.250 |
| BMI | 0.805 | 1.242 | - | - | 0.805 | 1.242 |
| MMSE | 0.822 | 1.216 | - | - | 0.822 | 1.216 |

VIF: variance inflation factor. HY, Hoehn and Yahr stage; LED DA, levodopa equivalent dose of dopamine agonist; HAMD, Hamilton Depression Scale; BMI, body mass index; MMSE, Mini‐Mental State Examination.
